# The application of metaverse in mental health

**DOI:** 10.3389/fpubh.2025.1463494

**Published:** 2025-04-10

**Authors:** Yue Wang, Boshi Duan, Xi Chen, Yuxuan Song, Xin Liu

**Affiliations:** ^1^Department of General Surgery, Cancer Hospital of China Medical University, Liaoning Cancer Hospital and Institute, Shenyang, China; ^2^Department of Medical Oncology, National Cancer Center/ National Clinical Research Center for Cancer/Cancer Hospital and Shenzhen Hospital, Chinese Academy of Medical Sciences and Peking Union Medical College, Shenzhen, China; ^3^Department of Clinical Integration of Traditional Chinese and Western Medicine, Liaoning University of Traditional Chinese Medicine, Shenyang, China; ^4^Pharmaceutical Science, China Medical University-The Queen’s University Belfast Joint College, China Medical University, Shenyang, China; ^5^Department of Colorectal Surgery, Cancer Hospital of China Medical University, Liaoning Cancer Hospital and Institute, Shenyang, China

**Keywords:** metaverse, mental health, artificial intelligence, big data, virtual reality

## Abstract

Rapid technological progress is reshaping human existence globally. The metaverse, a 3D digital realm merging virtual reality (VR) with physical space, exemplifies this fusion. Users can replicate and customize real-world elements within this immersive environment. Over the past decade, VR, augmented reality (AR), and mixed reality (MR) have become effective tools for addressing mental health conditions, offering solutions to the shortage of mental health professionals and limited access to care. However, extensive participation in 3D immersive gaming and social media can lead to insecurity, anxiety, depression, and addictive behaviors, particularly among young adults. This engagement may also impair attention spans, exacerbating symptoms in adolescents with attention deficit hyperactivity disorder. This research examines the impact of expanding metaverse applications on mental health, exploring both risks and benefits.

## 1 Introduction

The term “metaverse” refers to a collective, immersive, and interconnected digital universe that merges aspects of virtual reality (VR), augmented reality (AR), and mixed reality (MR) into a unified experience. It is a virtual environment where users interact with each other and digital objects in real-time, often through avatars or digital representations of themselves. This digital space allows users to engage in activities that blend both the physical and virtual realms, facilitating communication, entertainment, social interactions, and commerce. The content and interactions within the metaverse are diverse and continue to evolve. These can be broadly categorized into immersive virtual environments, user-generated content, social interactions, and economic exchanges. Virtual environments within the metaverse allow users to engage with a blend of the real and the virtual, often through avatars or digital representations of themselves. These avatars serve as a means for individuals to interact socially, carry out tasks, and even build virtual businesses. The content, such as games, virtual real estate, and digital art trading, contributes significantly to the complex social dynamics observed within the metaverse. The ability for users to create and customize their virtual spaces and identities adds a layer of personalization that can deeply affect their psychological well-being. Interaction within these spaces is conducted both asynchronously (e.g., through user-generated content like virtual art or games) and synchronously (e.g., real-time socializing in virtual chat rooms or multiplayer games), offering a unique blend of connection and immersion.

In spite of limited immersion and interactivity, VR fundamentals have existed for more than five decades (1). Immersive VR is enabled by head-mounted devices that incorporate stereoscopic displays and motion-tracking lenses, thus creating a compelling illusion of physical presence within a virtual realm (2). VR replaces the real world with a simulated counterpart, while AR enhances tangible surroundings with digital overlays, superimposing virtual imagery onto physical entities (3). As a result, VR creates a psychological immersion, fostering an illusory environment of virtual presence, while AR provides a platform for users to interact with virtual elements in the physical world (4). MR combines virtual and real realities, allowing seamless interaction between real and virtual elements and giving users greater control over virtual constructs compared to AR (5). In speculative literature, the metaverse represents the transformation of the Internet into a virtual realm through VR and AR (6), facilitating social and economic interactions via interconnected virtual spaces (7). Driven by immersive VR technologies and Web 3.0, a decentralized internet (8, 9), it presents challenges such as data privacy, user dependency, and well-being, mirroring issues in social media and gaming. Mental health issues, the leading cause of global disability (10), are compounded by stigma, limited resources, and work conflicts (11). The metaverse integrates social media, virtual games, e-commerce, and digital art trading, impacting mental health with both positive and negative effects (12, 13). Amara’s law suggests that new technologies may have underestimated long-term effects (14). This review explores the metaverse’s impact on mental health epidemiology.

In addition to its applications in entertainment, education, and mental health, the industrial metaverse has emerged as one of the most developed and rapidly growing sectors of the metaverse. This sector is focused on leveraging the metaverse to optimize industrial operations, enhance product design, and improve manufacturing processes. For instance, industries such as automotive, construction, and healthcare have begun integrating VR, AR, and MR technologies into their workflows. These applications help in training employees, simulating real-world environments for testing purposes, and facilitating remote collaboration in manufacturing plants. The industrial metaverse also offers improved user experiences (UX) by providing more practical and immersive environments for real-time collaboration and decision-making. Unlike the gaming or social metaverse, which primarily focuses on entertainment and socializing, the industrial metaverse focuses on practical applications, offering a more functional experience aimed at improving productivity and reducing costs. Its implementation varies significantly from that of other digital environments due to its specific focus on real-world industrial applications and its emphasis on efficiency and optimization. This review explores the impact of metaverse applications on mental health interventions, focusing on VR, AR, and MR. It covers current uses in exposure therapy, rehabilitation, and the treatment of conditions like PTSD, depression, and phobias. The review also discusses future directions, stressing the need for technological advancements, better biomarkers, and AI-driven interventions. Lastly, the review calls for more longitudinal studies and clinical trials to validate the effectiveness of metaverse applications in mental health care.

## 2 Current applications in mental health

### 2.1 Exposure therapy

VR technology offers significant potential for exposure therapy, allowing safe confrontation of traumatic stimuli in virtual environments without real-world exposure. It overcomes the challenge of replicating trauma intensity through immersive sensory experiences that enhance realism while ensuring safety (15). One example is “Virtual Iraq,” a PTSD treatment system where patients interact with virtual recreations of Iraq and Afghanistan, showing over 80% of participants no longer meeting PTSD diagnostic criteria post-treatment (16). VRET is particularly effective for specific phobias, like fear of small animals, and has also shown promise for public speaking and claustrophobia (17, 18). A study with 9/11 survivors showed sustained therapeutic gains 6 months after exposure (19). VRET, when combined with traditional therapies, offers additional benefits (20). Increased public acceptance of VR as a realistic tool has led to more individuals seeking treatment (21, 22). VRET is also cost-effective, offering better efficiency and ROI compared to *in vivo* therapies. Cost-effective home-based VRET systems, such as those developed by Krzystanek et al. (23), allow personalized treatment and reduce stigma. Despite uncertainties about long-term outcomes, VRET shows promise for phobia treatment (24).

### 2.2 Virtual rehabilitation

Virtual rehabilitation uses VR technology and computer simulations to provide therapeutic exercises and interventions. This approach enhances traditional rehabilitation by creating immersive, interactive environments that engage patients. It can be applied to various conditions, including physical therapy for movement disorders, cognitive rehabilitation for brain injuries, and mental health therapy. VR enables personalized, motivating, and effective treatment options accessible in clinical settings or at home. In psychology, virtual rehabilitation denotes a therapeutic approach wherein a patient’s training primarily relies on, or is supplemented by, simulated experiences within virtual reality (25). When conventional therapy is absent, the rehabilitation is termed “virtual reality-based”; whereas, if virtual rehabilitation is administered alongside traditional therapy, it is referred to as virtual reality-augmented. With the pervasive integration of virtual environments into daily life. Virtual rehabilitation and gaming-based rehabilitation, which uses gaming consoles to conduct rehabilitation, are increasingly popular because a quarter of the world’s population has access to the internet (26). Notably, diverse disorders can be managed more effectively with virtual therapy than with traditional therapies.

### 2.3 Depression

In the year of 2006, the UK issued recommendations advocating for the integration of Virtual Reality Therapy (VRT) within the National Health Service across England and Wales. This recommendation particularly emphasized the utilization of VRT as an alternative to immediate antidepressant medication for patients presenting with mild to moderate depression (27). Certain regions have undertaken initiatives to implement or trial such recommendations. At Auckland University in New Zealand, Dr. Sally Merry and her team have spearheaded the development of a computerized Cognitive Behavioral Therapy (CBT) fantasy “serious” game aimed at addressing adolescent depression. This game, named Sparx, boasts a multitude of features designed to combat depression. Through Sparx, users immerse themselves in a fantasy realm, assuming the role of a character who navigates challenges by directly confronting negative thoughts and acquiring techniques for depression management (28).

### 2.4 Eating disorders and body dysmorphia

There is growing evidence that virtual reality therapy can be used to treat eating disorders and body dysmorphia (29). A noteworthy investigation undertaken in 2013 involved participants in a sequence of activities within virtual reality environments, tasks that would have been difficult to replicate without the assistance of sophisticated technology. During the program, patients were shown what happened when they reached their goal weight, compared their real body shape to their perceived size, and adjusted their virtual image to reflect their actual body proportions (30).

### 2.5 Gender dysphoria

Initial studies suggest that VR encounters show potential in providing therapeutic alleviation to transgender individuals struggling with gender dysphoria (31). Yet, extensive experimentation and professional scrutiny are imperative prerequisites before VR could be considered a viable clinical intervention. Nevertheless, anecdotal evidence suggests that some transgender individuals have resorted to self-administered virtual sex reassignment therapy, albeit informally. Within digital realms, individuals find avenues for anonymous self-expression, a privilege often denied to them in real-life settings due to pervasive discrimination and violence. The sophistication of VR technology amplifies these newfound freedoms by furnishing a platform with gender dysphoria to embody gender identities, especially when such expression is constrained offline. Leveraging available VR applications, including video games and virtual chat spaces, individuals can craft avatars, engage anonymously, and pursue therapeutic objectives (32).

### 2.6 Acrophobia

Acrophobia is an intense fear of heights, often causing significant anxiety and avoidance behaviors (33). Phobia can lead to physical symptoms such as dizziness, sweating, and palpitations when exposed to high places. Anxiety associated with this condition can be managed through cognitive-behavioral therapy, exposure therapy, and sometimes medication (34). There is evidence that VR therapy can effectively treat acrophobia (35, 36). During these study, participants encountered challenging heights within a virtual setting and were assigned diverse tasks at these elevations. While the study’s breadth and depth may not fully warrant immediate integration into clinical protocols, its findings offer a hopeful trajectory for future research and therapeutic frameworks, particularly given that a significant portion of participants reported a reduction in their fear of heights (37).

### 2.7 Stroke

Research shows stroke patients benefit significantly from VR rehabilitation in their physical therapy regimens (38). After rehabilitation to restore balance and walking, survivors often need to relearn muscle control, typically through rigorous, repetitive exercises that are physically taxing and slow in yielding results. In contrast, VR-augmented therapy offers personalized training tailored to each patient’s needs (39). While exercises remain repetitive, VR enhances engagement through diverse virtual environments, improving motor learning and real-world performance. Feedback, especially visual feedback, plays a crucial role in accelerating motor learning and improving balance recovery (40). Research also indicates that VR training leads to better walking speed improvements compared to traditional therapy (41). A recent review showed that VR-based training, when matched in duration and dosage to conventional therapy, significantly improved gait velocity and Berg Balance Scale assessments (38).

### 2.8 Parkinson’s disease

In Parkinson’s disease, movement is affected by tremors, stiffness, slowness, and balance problems. Although there is no cure, treatments such as medication, physical therapy, and deep brain stimulation can relieve symptoms and improve quality of life (42). The use of VR technology in physical therapy interventions for Parkinson’s patients has been demonstrated to be beneficial in several studies (43). The observed improvements are attributed to the enhanced feedback provided to patients during VR sessions. VR has been shown to engage and stimulate both motor and cognitive processes in patients, which are often affected by the disease. Furthermore, the replication of real-life scenarios in VR enables patients to practice functional activities, contributing to their rehabilitation progress (44).

### 2.9 Wound care

Furthermore, the implementation of VR in wound care rehabilitation has demonstrated favorable outcomes. Studies suggest that the level of immersion in VR correlates with increased engagement and focus of patients on the virtual environment (45). Importantly, VR has demonstrated efficacy in reducing pain, anxiety, and depressive symptoms, while simultaneously enhancing adherence to treatment protocols. Other research indicates that VR provides distraction, leading to reduced time spent dwelling on pain and lower pain intensity, fostering immersion and facilitating procedures (46). Pain is frequently associated with wound dressing procedures. Thus, the adoption of VR has been linked to improved dressing efficiency, heightened distraction from pain during tasks like dressing changes and physical rehabilitation, consequently mitigating patients’ stress and anxiety.

### 2.10 Autism

Autism is a developmental disorder affecting communication, behavior, and social interactions (47). The cause is believed to involve genetic and environmental factors. VR has emerged as a promising method for improving social skills in young adults affected by autism spectrum disorders (ASD) (48). Participants engaged with a virtual avatar within a range of simulated environments, navigating through social scenarios including interviews, interpersonal interactions, and conflict resolution tasks. Results revealed significant advancements in emotional perception, encompassing both vocal and facial cues, alongside heightened abilities in perspective-taking. Notably, participants provided overwhelmingly positive feedback regarding the efficacy of the interventions during follow-up assessments conducted months after the study’s completion (49). Furthermore, numerous additional investigations have examined the potential of VR as an occupational therapy option for individuals with ASD.

### 2.11 Post-traumatic stress disorder

Post-traumatic stress disorder (PTSD) is a mental health condition triggered by experiencing or witnessing a traumatic event (50). VR could be a valuable tool to help those with Post-Traumatic Stress Disorder (51). Through VR technology, patients can revisit their traumatic experiences under controlled conditions, with therapists guiding them through varying intensities of exposure. Certain experts advocate for this approach, asserting its efficacy in PTSD treatment by enabling the faithful recreation of past events. As one scholar notes, it facilitates heightened patient engagement and, consequently, enhanced activation of traumatic memories, a crucial element for fear extinction (52).

### 2.12 Chronic and acute pain

In adults and children with a variety of medical conditions, immersive VR therapy has been investigated as a supplementary pain treatment (53). While conventional treatment modalities have typically included pharmacological interventions and physical therapy, their long-term efficacy is constrained by financial implications and adverse reactions (54). However, Research on its efficacy in managing chronic pain has been limited. Notably, VR has been found to be effective in alleviating chronic pain without causing headaches, dizziness, or nausea in chronic pain sufferers (55).

### 2.13 Neurological rehabilitation

VR has proven effective for addressing balance and mobility issues from strokes or head injuries (56). While recent studies suggest a slight advantage over traditional training, larger randomized trials are needed to explore video-capture VR and its combination with standard therapy (57). Follow-up assessments show improved mobility and balance for individuals with cerebral palsy (CP) after VR interventions (58). In pediatric CP therapy, VR enhances balance, gait, and function alongside other treatments like physical therapy and medication (59). VR has also improved upper limb function and postural control, especially in younger patients due to greater neuroplasticity (60). Gamification and supportive virtual environments boost motivation and attention, promoting task repetition for neurological changes (60). Functional MRI studies suggest VR induces neuroplastic changes in the sensory motor cortex, improving motor function (61). Collaborative development of VR therapies and provider training improves outcomes. Customized VR systems tailored for specific therapeutic goals offer more effective engagement and feedback compared to off-the-shelf platforms (62). By simulating real-life task complexities, VR aids skill transfer and adaptive strategies, improving treatment outcomes (63).

## 3 The mechanism of metaverse advancing mental health interventions

The metaverse holds significant potential for advancing mental health interventions. First, it provides an opportunity to enhance accessibility. Virtual platforms enable individuals who face barriers such as geographic isolation, limited mobility, or stigma-associated fear of seeking help to access mental health resources discreetly and conveniently. These tools can ensure a continuity of care regardless of location, bridging gaps in regions with insufficient mental health professionals (64). Social presence, a key determinant of therapeutic engagement, is amplified in multi-user virtual environments. For instance, Wienrich et al. (65) demonstrated that social interdependence in location-based VR fosters cooperation and shared emotional experiences, enhancing treatment adherence and group dynamics. Similarly, Matsangidou et al. (66) designed a multi-user VR psychotherapy platform for body weight concerns, where participants reported heightened self-awareness and reduced stigma through real-time peer interactions. These studies underscore how social presence in shared virtual spaces can replicate—and even augment—the relational dynamics of in-person therapy, creating a sense of collective support critical for conditions like anxiety and eating disorders. Second, the metaverse can facilitate highly personalized and adaptive therapeutic experiences. With the aid of artificial intelligence, interventions can be tailored to match an individual’s clinical profile, symptom severity, and progress (67). For instance, interactive virtual simulations can expose patients to controlled environments designed for specific therapeutic goals—such as gradually confronting anxiety-provoking situations in a safe and supervised manner (17). This element of controlled exposure therapy is particularly beneficial for treating conditions like phobias, post-traumatic stress disorder (PTSD), and various anxiety disorders (68). Third, the social connectivity enabled by the metaverse can foster supportive communities. Virtual support groups and peer communities allow individuals to share experiences and coping strategies in real-time, reducing feelings of isolation and stigma. Mental health professionals can also lead group sessions or workshops, further normalizing help-seeking behaviors (69). Fourth, advanced data analytics in metaverse environments can assist clinicians in tracking user interactions, psychological states, and treatment adherence. Biometrics, voice modulation, and facial expression analysis can provide objective insights into a patient’s mental state, enabling early detection of relapses or treatment plateaus (70). This continuous feedback loop helps healthcare providers optimize interventions and improve patient outcomes. Finally, integrating gamification elements in the metaverse can enhance patient engagement. Gamified modules can break down complex therapeutic exercises into interactive, more enjoyable tasks. This increased engagement often correlates with better adherence, greater emotional resilience, and more sustainable long-term improvements (71).

The metaverse has the potential to redefine mental health interventions by merging therapeutic techniques, social engagement, and individualized care into a single immersive experience. Unlike traditional approaches, which may separate therapeutic interventions from social interactions, the metaverse offers a unified space where individuals can receive therapy, engage in supportive communities, and experience emotional growth. Through the use of avatars and virtual environments, users can confront psychological challenges, receive personalized care, and develop coping strategies within a virtual ecosystem that adapts to their evolving needs. This interconnectedness of therapeutic, social, and personal spaces is a unique contribution of the metaverse, offering a more holistic approach to mental health care.

## 4 Projected benefits on mental health

### 4.1 Social connectedness

Interpersonal connections in the metaverse offer a unique way to build relationships, transcending geographical limits and allowing engagement in communities with shared interests or identities. Marginalized populations and those facing mental health challenges can particularly benefit, gaining peer support and knowledge exchange (72). Recent advancements in immersive multi-user VR platforms highlight the role of social presence in mitigating loneliness. For example, Fajnerova et al. (73) systematically reviewed the transition from nonimmersive to immersive multiuser mental health applications, emphasizing that shared virtual environments with high social presence (e.g., avatars with realistic nonverbal cues) improve emotional resonance and reduce feelings of isolation. Similarly, Matsangidou et al. (66) found that collaborative tasks in VR, such as joint problem-solving or role-playing, strengthened participants’ social self-efficacy and body acceptance through mutual feedback. A global survey of online role-playing game players found strong relationships, with some developing into real-world friendships or romantic connections (74). Virtual environments show promise in fostering new connections and strengthening existing ones (75). Further research is needed to understand the impact of these social dynamics on mental well-being, particularly in alleviating loneliness and enhancing social connectivity.

### 4.2 Self-determination

In virtual environments, self-determination can be achieved through experiences that promote autonomy and competence. A survey of 672 players in massively multiplayer online role-playing games revealed positive outcomes like accomplishment, excitement, manageable stress, communal engagement, and increased self-assurance (76). Similarly, studies with college students showed that autonomy and competence during gameplay were linked to higher motivation and enjoyment (77). For individuals with disabilities or mental health struggles, digital realms offer spaces where psychological needs for self-determination can be met.

### 4.3 Self-representation

The metaverse’s virtual environments impact users’ mental well-being due to their configurability, allowing personalization of settings (78). This autonomy enables VR relaxation, which may reduce anxiety and improve mood in the short term (79). However, virtual environments can also cause distress, particularly in social contexts. Customizing avatars affects perceptions and interactions, with studies showing that increased avatar embodiment reduces awareness of physical sensations. Altering avatar height during VR experiences can increase paranoia and negative social comparisons, especially among women. While virtual identity experimentation may aid adolescent social skills, excessive focus on self-representation can lead to body dissatisfaction, similar to social media effects.

### 4.4 Physical activity

The metaverse offers opportunities to stimulate cognitive function, as many games are designed to challenge players’ cognitive abilities through problem-solving tasks. A study found that adults with major depressive disorder showed significant improvements in attention and cognitive function through video game-based interventions compared to an active control group (80). Additionally, navigating virtual environments using motion-tracking sensors can promote physical activity, and games incorporating physical activity have been shown to enhance cognitive functioning in both clinical and nonclinical settings (81).

### 4.5 Work-life balance

Although primarily aimed at improving industrial operations, the industrial metaverse may have indirect benefits on mental health by reducing work-related stress, enhancing employee training, and promoting work-life balance. For example, VR training modules can simulate high-stress environments, allowing employees to practice and refine their skills in a safe and controlled setting, thus reducing real-world anxiety. Additionally, remote collaboration tools within the industrial metaverse may provide employees with more flexible working conditions, potentially alleviating stressors related to long commutes or rigid office hours. However, the demands of a high-tech industrial metaverse may also introduce new challenges, such as work-related burnout, isolation, and the potential for over-reliance on digital systems.

### 4.6 Multiuser engagement

Multiuser metaverse environments provide new opportunities for mental health interventions by fostering real-time, multidimensional interactions that enhance social connectedness and promote interdisciplinary collaboration. These platforms can simulate group therapy or support groups, allowing participants to share experiences and coping strategies in an immersive, supportive atmosphere. For those unable to attend in-person sessions due to stigma or geographic barriers, the anonymity of the metaverse reduces judgment, encouraging more people to seek help. The immersive nature allows immediate feedback from professionals and peers, improving the timeliness and personalization of interventions. Experts from various fields can collaborate on the same platform to create comprehensive plans, providing both professional guidance and peer support essential for long-term recovery and growth.

## 5 Projected negative effects on mental health

### 5.1 Addictions

Virtual worlds, by providing repetitive rewarding experiences, may foster addiction-like behaviors. Gaming disorder, characterized by excessive gaming, impaired control, and neglect of other activities, affects 2–3% of the global population, with online gaming being more prevalent (82). Although the issue of problematic gaming is debated, evidence suggests it negatively impacts some individuals (83). The metaverse, with its immersive technology, can lead to deep virtual immersion, disrupting real-world functions and increasing addiction risks. Companies may exploit user behavior through passive monitoring and tailored environments to enhance engagement (84).

### 5.2 Circadian rhythms

Excessive involvement in the metaverse may displace offline activities, negatively affecting mental health by reducing time spent on beneficial activities (85). Notably, participation in virtual worlds, particularly in the evenings, may disrupt sleep patterns, leading to shorter or interrupted sleep. A review suggests that adolescents’ poor sleep outcomes are linked to increased screen time (86), although experimental studies have not consistently proven a causal relationship. Three potential mechanisms for screen-related sleep disturbances are: light emission from screens, delayed sleep initiation, and psychological arousal from digital media content (86). The metaverse’s immersive nature may further exacerbate these effects.

### 5.3 Stress exposure

People often use virtual environments to escape or cope with real-world stress. Surveys suggest that avoidance and escapism are primary motivations for engaging in video games. Participants reported using gaming to avoid life’s challenges rather than confronting them directly. While repeated avoidance may hinder resilience, video games can also serve as an adaptive coping mechanism, aiding mood regulation after stress (87). A study of video gamers found escapism linked to both positive and negative outcomes. Further research is needed to explore how escapism in the metaverse affects mental well-being.

### 5.4 Interactions

A key issue is how the shift from face-to-face to digital social interactions affects mental well-being. Studies show that online gaming fosters social interactions, both in and out of virtual spaces. However, increased engagement in the metaverse may reduce real-world interactions, potentially worsening social anxiety and interpersonal skills. This could explain the link between internet addiction and feelings of isolation. For individuals with social anxiety or limited social skills, virtual worlds may serve as both a compensatory measure and a barrier to in-person social engagement.

### 5.5 Multiuser social interactions—potential pitfalls

While multiuser metaverse environments offer expanded opportunities for mental health support, they also present risks that need careful attention. The lack of face-to-face interaction may hinder the development of real-world social skills, especially for those with social anxiety, leading to overreliance on virtual interactions and difficulty recognizing emotional cues offline. The high level of immersion can also amplify the spread of negative emotions within groups, where one member’s distress can quickly affect others, increasing stress across the community. Additionally, cyberbullying and anonymous aggression are significant concerns, as the anonymity of virtual spaces may encourage harmful behaviors, further exacerbating anxiety or depression, particularly for vulnerable users. To ensure safe and constructive engagement, developers and clinicians must implement effective behavioral guidelines and supervision in these virtual spaces.

## 6 Challenges

A thorough evaluation of metaverse applications in mental health requires prolonged observation. As the Metaverse becomes more integral to our lives, it brings both new challenges and opportunities for social interaction and education. Reality and identity issues are key challenges, as the ability to create different avatars can confuse younger users still forming their self-identity, potentially causing psychological distress due to blurred boundaries between virtual and real identities (88). Drawing on cross-disciplinary experiences from industries such as tourism, we can develop effective psychological health interventions in the metaverse by integrating best practices, insights, and strategies that have proven successful in other fields. By borrowing strategies and best practices from tourism, we can develop more engaging, personalized, and effective psychological health interventions in the metaverse, creating a virtual ecosystem where users can improve their well-being in a safe, supportive, and innovative way. While social presence enhances therapeutic engagement, its design and ethical implementation remain challenging. For instance, Wienrich et al. (65) noted that poorly calibrated social interdependence in VR could inadvertently amplify social anxiety or group conflict. Similarly, Fajnerova et al. (73) cautioned that immersive multiuser environments may exacerbate identity distortion if users prioritize idealized virtual personas over authentic self-expression. Balancing social presence with psychological safety is critical to avoid unintended harm. Global connectivity in the Metaverse can lead to social isolation due to shallow virtual interactions (89). Anonymity enables cyberbullying, worsening anxiety and depression (90). Addictive content may neglect real-life duties, exacerbating mental health issues (91). Large-scale data collection raises privacy concerns, increasing anxiety and vulnerability (92). Next, it should be noted that the analysis of the metaverse’s impact on mental health remains speculative. As the metaverse is still largely in development, its long-term effects, both positive and negative, are yet to be fully understood. The current review is based on the existing literature and early applications of VR, AR, and MR technologies, but these insights are subject to change as the metaverse matures and becomes more integrated into daily life. Additionally, the individual and social dynamics of the metaverse are complex, and more longitudinal studies and clinical trials are needed to assess their true impact on mental health. Last, the metaverse is poised to not only transform how we deliver mental health care but also how we understand and experience psychological well-being. By integrating advanced AI, biometric tracking, and real-time social support, the metaverse can create adaptive, personalized therapeutic experiences that respond dynamically to users’ emotional and psychological states. However, this potential requires a deeper exploration of how such systems will function at scale, ensuring that interventions are evidence-based, accessible, and ethical. Longitudinal studies and cross-disciplinary research are necessary to fully understand the metaverse’s long-term impact on mental health, including its potential to enhance or hinder individual well-being.

## 7 Limitations

### 7.1 Ethical concerns

The metaverse raises ethical concerns about privacy, data security, anonymity, inequitable access, and identity distortion. Personal data collection, including biometrics, poses privacy risks and misuse, such as identity theft or harm. Anonymity can fuel cyberbullying, worsening anxiety and depression. High VR/AR costs limit access, deepening inequalities in mental health support. Avatars differing from real identities may cause distress, especially in younger users. These issues stress the need for frameworks to protect users’ safety and well-being. Blockchain technology offers a robust framework for enhancing data security and privacy within virtual environments, such as the metaverse. Its inherent characteristics-decentralization, immutability, transparency, and the use of cryptographic protocols-address several key vulnerabilities associated with traditional centralized systems.

### 7.2 Social isolation

While virtual environments can facilitate connections, the shift from face-to-face interactions to digital engagement may erode interpersonal skills and intensify feelings of loneliness. This paradox—where increased virtual connectivity leads to greater real-world isolation—raises concerns, particularly for individuals already vulnerable to social anxiety. The metaverse may also encourage users to replace real-world relationships with virtual ones. While online interactions can be meaningful, they often lack the emotional depth and richness of physical interactions, leaving users feeling disconnected and unsupported. Additionally, excessive use of virtual environments, especially among adolescents, may hinder the development of critical social and emotional skills, limiting their ability to effectively navigate real-world challenges.

### 7.3 Overreliance on virtual environments

Overreliance on virtual environments, especially in the metaverse, raises concerns about addiction-like behaviors, with users prioritizing virtual engagement over real-world activities and neglecting responsibilities. Prolonged use of VR headsets and digital devices can lead to physical issues like eye strain, headaches, and musculoskeletal problems, alongside psychological effects such as increased stress and anxiety. Virtual spaces can also foster dissatisfaction with real-life settings, creating unrealistic expectations and contributing to cognitive dissonance. Excessive use, particularly in the evenings, may disrupt circadian rhythms, affecting sleep and overall mental health.

## 8 Conclusion

While much of the current research in the metaverse focuses on individual technologies such as VR, AR, and MR, the metaverse as a collective and immersive digital space holds a unique potential to reshape the paradigm of mental health interventions. Unlike traditional therapies, the metaverse creates a fully interconnected environment that can blend various aspects of therapy, social support, and daily living into one seamless experience. This paradigm shift offers the opportunity to explore mental health through novel, immersive social interactions, the personalization of therapeutic spaces, and more adaptive, continuous support systems that transcend geographic and temporal limitations. The metaverse allows users to engage with mental health resources not just as passive recipients, but as active participants in an evolving virtual ecosystem that can address both psychological and emotional well-being, offering a much-needed shift from conventional interventions that may not fully address the holistic needs of individuals. Mental health services are experimenting with the metaverse approach based on preliminary studies. This framework explores the brain’s potential in social interactions for treating cognitive and emotional disorders. However, significant advancements in methodology and clinical trials are needed to fully utilize these technologies. The usability of new devices and the application of AI algorithms in metaverse tools should be assessed before new clinical studies begin. Machine learning models can analyze human actions, but require plausible biomarkers to correlate with clinical scores, similar to how robotic metrics are used in neurorehabilitation.
